# The Prognostic and Therapeutic Potential of Fragile X Mental Retardation 1 (*FMR1*) Gene Expression in Prostate Adenocarcinoma: Insights into Survival Outcomes and Oncogenic Pathway Modulation

**DOI:** 10.3390/ijms25137290

**Published:** 2024-07-02

**Authors:** Salem Baldi, Bushra Amer, Fawze Alnadari, Maged AL-Mogahed, Yaqin Gao, Yaser Gamallat

**Affiliations:** 1 Department of Medical Laboratory Diagnostics, School of Medical Technology, Shaoyang University, Shaoyang 422000, China; 2 Department of Family Medicine, Michigan State University, East Lansing, MI 49684, USA; bushraamer2014@gmail.com; 3 Research and Development Center of Jiangsu Tianmeijian Nature Bioengineering Co., Ltd., Nanjing 210046, China; 2019208038@njau.edu.cn; 4 Department of Surgery, The First Bethune Hospital of Jilin University, Changchun 130012, China; al22@mails.jlu.edu.cn; 5 Department of Ultrasound Diagnosis, Zhongda Hospital Southeast University, Nanjing 210009, China; 6 Department of Oncology, Biochemistry and Molecular Biology, Cumming School of Medicine, University of Calgary, Calgary, AB T2N 1M4, Canada

**Keywords:** *FMR1*, PRAD, gene expression, correlation, *PI3K_AKT_mTOR*

## Abstract

Prostate adenocarcinoma (PRAD) is the second most common tumor associated with death. The role and mechanisms of the fragile X mental retardation 1 (*FMR1*) gene in PRAD remain unknown. We conducted an analysis of *FMR1* expression in PRAD to determine its prognostic importance and connection to carcinogenic pathways such as *PI3K_AKT_mTOR*. Survival analyses were utilized to establish a correlation between *FMR1* expression and patient outcomes. We used the integration of genomic data with bioinformatic predictions to predict the regulatory factors of the *FMR1* gene in PRAD. Our data revealed that individuals with higher levels of *FMR1* expression experience worse survival outcomes compared to those with lower expression (hazard ratio [HR] = 5.08, 95% confidence interval [CI] = 1.07 – 24, *p* = 0.0412). *FMR1* expression was significantly higher in patients with advanced pathological tumor stages, particularly in the pT3 and pT4 combined stages and the pN1 nodal stage. Furthermore, patients with high Gleason scores (GSs) (combined GSs 8 and 9) exhibited increased levels of *FMR1* expression. Our results further identify a possible regulatory link between *FMR1* and key oncogenic pathways, including *PI3K_AKT_mTOR*, and predict the possible mechanism by which *FMR1* is regulated in PRAD. Our data suggest that the *FMR1* gene could serve as a biomarker for PRAD progression. However, in-depth investigations, including those with large patient samples and in vitro studies, are needed to validate this finding and understand the mechanisms involved.

## 1. Introduction

PRAD is the second most common tumor associated with death. While screening and treatment for this type of tumor have made some progress in recent years, the molecular mechanisms involved in its progression remain largely unknown [1].

The fragile X mental retardation 1 (*FMR1*) gene was originally identified as a key player in fragile X syndrome [2]. It encodes the fragile X mental retardation protein (FMRP), which is critical for synaptic plasticity and neuronal development [3,4]. However, recent studies have revealed its involvement in various health issues, including cancer [5].

The loss or mutation of *FMR1* leads to a variety of developmental and neurological issues, underscoring its importance in normal brain function. The *FMR1* genotype affects synaptic connectivity, indicating the importance of FMRP in synapse formation and maintenance [6]. Another study found that the translation enhancement induced by the depolarization of nerve cells is mediated by the phosphorylation of the YTHDF1-binding protein FMRP [7,8]. These findings suggest that *FMR1* may play a crucial role in regulating cellular responses that are essential in cancer [9].

It is well known that RNA-binding proteins can influence cancer progression, which could provide a framework for understanding *FMR1*’s potential roles in tumorigenesis [10]. On the other hand, *Enokida* et al. found a significant epigenetic modification in cancer progression involved *FMR1*’s expression as a potential regulator of cancer [11]. Circular RNAs (CircRNAs) act as miRNA sponges, regulating the availability and activity of miRNAs. Researchers reported that hsa_circ_0037858 regulates the activity of miR-5000-3p, which, in turn, controls the expression of the *FMR1* gene, a protein associated with the spread of clear cell renal cell carcinoma [12]. Further evidence suggests that miR-323a-3p enhances clinical outcomes for patients with esophageal squamous cell carcinoma (ESCC) by inhibiting *FMR1* [13].

The *PI3K/AKT/mTOR* pathway is essential in the biology of PRAD as it controls multiple cellular activities, including proliferation, survival, metabolism, and drug resistance. Genetic changes such as *PTEN* deletion frequently activate this pathway, promoting tumor development and advancement, leading to a negative prognosis and treatment difficulties [14,15]. Another study also highlights the importance of the pathway in controlling apoptosis, cell proliferation, metastasis, and invasion in PRAD and explores the connection between the *PI3K/Akt/mTOR* pathway and tumorigenesis [16]. The pathway’s prominence in PRAD is underscored by the finding that it is upregulated in 30–50% of cases, often due to genetic alterations identified in Genome-wide association studies (GWASs) [14].

A recent study found that *FMR1* is upregulated in colorectal cancer (CRC) and promotes proliferation and metastasis [5,15]. Its expression patterns in other cancer types have suggested potential involvement in oncogenesis. Ding et al. identified *FMR1* as one of seven N6-methyladenosine (m^6A) RNA methylation-related genes that act as cross-talk genes between PRAD and periodontitis (PD), emphasizing *FMR1*’s prognostic relevance in PRAD [17]. *FMR1* has the potential to influence the infiltration of immune cells into the tumor microenvironment and the clinical prognosis of human cancer [18,19]. This insight may pave the way for further research on *FMR1* as a target for therapeutic intervention and a marker for prognosis in PRAD. Nevertheless, the role and mechanisms by which *FMR1* controls PRAD remain unknown.

In the current study, we hypothesize that the *FMR1* gene may contribute to the aggressiveness of PRAD and modulate the efficacy of therapeutic interventions. This study aims to assess *FMR1* expression levels in PRAD patients using TCGA PRAD data and examine their association with disease progression.

## 2. Results

### 2.1. Gene Expression Profile and Prognostic Implication of FMR1

**Figure 1:**
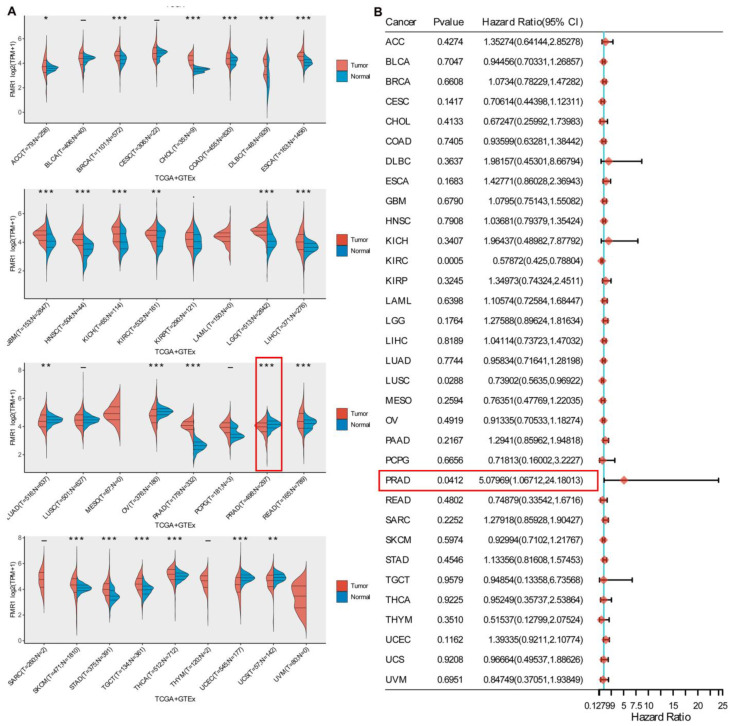
**Pan-cancer data assessment of *FMR1* gene expression and its prognostic relevance**. (**A**) Violin plots show the *FMR1* gene expression data distribution of tumors (red) and normal samples (blue). Among other cancers, *FMR1* expression is high in normal tissues compared to PRAD tissues (T = 498; N = 297). (**B**) The pan-cancer prognosis results of univariate Cox regression analysis. The ‘forest plot’ R package displays *FMR1*’s *p* value, HR, and 95% CI in each cancer in the forest plot. Distinct colors correspond to the functions of genes in various types of cancer. The color red and HR 5.07969 (95% CI (1.06712,24.18013) are used to indicate risk factors, indicating that greater *FMR1* gene expression is associated with a worse prognosis. Statistically significant differences between tumor and normal samples are indicated with asterisks above the plots (*** for *p* < 0.001, ** for *p* < 0.01, and * for *p* < 0.05).

We conducted a comprehensive investigation of the *FMR1* gene in Pan-cancer, and the findings revealed that its expression profiles exhibited variability. The violin graphs in Figure 1A demonstrate substantial differences in *FMR1* gene expression between tumor and normal samples in several types of cancer. Remarkably, the alteration in its expression level in PRAD was statistically significantly low, with a *p* value of *p* < 0.001. The PRAD prognosis results of the univariate Cox regression analysis were statistically significant, with a *p* value 0.04. The hazard ratio was 5.07969, with a 95% confidence interval (CI) ranging from 1.06712 to 24 (Figure 1B). These data further highlighted the possible potential role of *FMR1* expression in PRAD and its relation to disease prognosis.

### 2.2. Expression of FMR1 Decreases Overall Survival in PRAD Patients

**Figure 2:**
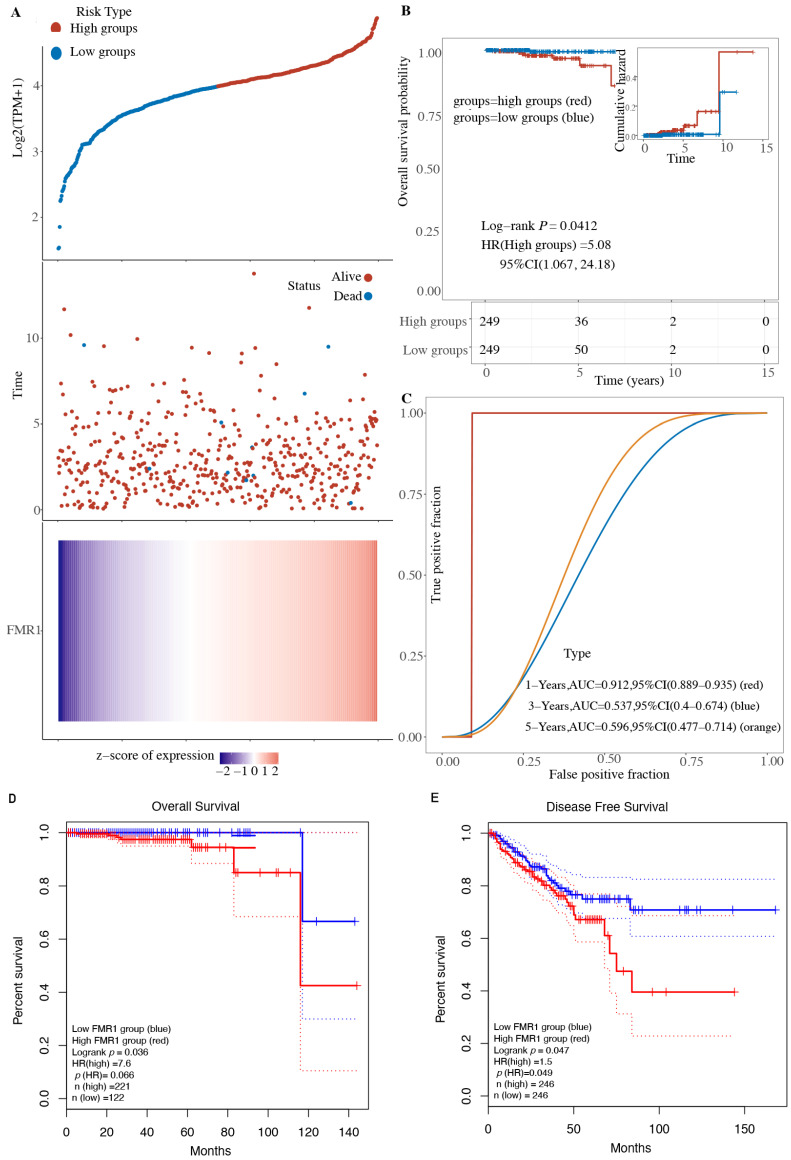
**Kaplan–Meier survival curves for patients with high vs. low *FMR1* gene expression**. (**A**) At the top of the image, scatter plot shows the survival rates of two groups, “high groups” and “low groups,” based on *FMR1* expression. The middle scatter plot shows the correlation between *FMR1* expression and patient survival status, and a heatmap shows in detail the variation in gene expression across samples in the bottom part. (**B**) Kaplan–Meier (KM) survival curves show how the risks are different for two groups of patients whose *FMR1* levels change over time. (**C**) ROC curves show that *FMR1* expression levels can predict patient outcomes over different time periods. It provides the AUC values for 1 year (0.912), 3 years (0.537), and 5 years (0.569), along with their corresponding 95% confidence intervals. (**D**,**E**) KM curves of patients’ data obtained from TCGA and analyzed using GEPIA demonstrate the effect of *FMR1* expression on patients’ outcomes.

We further examined the effects of the *FMR1* gene on the survival rates of a TCGA PRAD cohort. Our data show a potential correlation between elevated *FMR1* gene expression and reduced overall survival, which is associated with a less favorable survival outcome (Figure 2A). The log-rank test comparing the high and low groups yielded a *p*-value of 0.04, indicating a significant difference in the survival rates between the two groups. The hazard ratio (HR) for the high group is 5.08, indicating that the risk of the event occurring (often death or disease progression) is almost five times greater in the high group compared to the low group, as shown in Figure 2B. The high-risk category exhibits a diminished probability of survival. Figure 2C uses ROC curves to show how *FMR1* expression levels can predict how long a patient will live at different time points. The ROC curve analysis is crucial to understanding the *FMR1* function in PRAD; the curve closer to the top-left corner signifies a higher level of accuracy in the test measurement. With an area under the curve (AUC) of 0.9 for the 1-year survival prediction curve based on *FMR1* expression level, the ROC curve accurately detects high-level positive expression of *FMR1* and reliably predicts unfavorable survival outcomes for patients under evaluation within a year. An AUC value of 0.9 does not imply perfect discrimination; rather, it indicates a significantly high level of discrimination. A value of 1.0 for the AUC would indicate perfect discrimination.

These Figures collectively indicate that high *FMR1* expression is associated with poorer survival outcomes in PRAD patients. The Kaplan–Meier plot shows a clear distinction in survival probability between the high and low expression groups, and the ROC curve supports the predictive power of *FMR1* expression for short-term survival.

### 2.3. FMR1 Expression Is Correlated with Advanced Pathological Stages in PRAD

**Figure 3:**
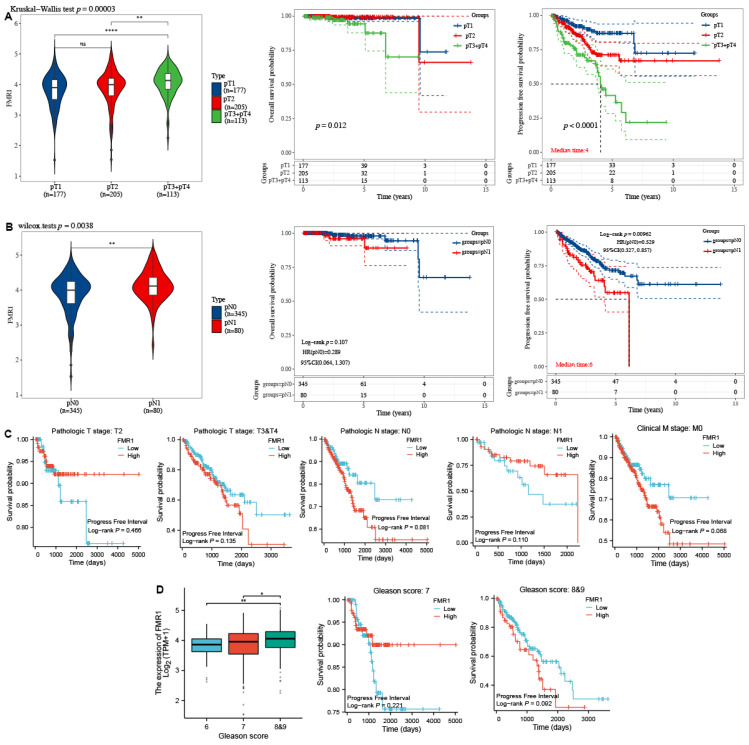
**FMR1 gene expression across different stages of PRAD.** (**A**) Violin plot displays the relationship between *FMR1* gene expression and the pathological T stages in PRAD (left side), and Kaplan–Meier survival curve shows overall survival and disease-free survival rates for patients in these stages (right side). (**B**) Violin plot displays the relationship between *FMR1* gene expression and the pathological N stages in PRAD (left side). It also displays the Kaplan–Meier survival curve that shows the overall survival and progression-free survival rates for patients in these stages (right side). (**C**) Data of *FMR1* expression of patients with different PRAD stages (pT2, combined pT3 and pT4, pN0, pN1, and M0) were split using best cutoff median to study its effect on outcomes (overall survival and progression-free survival). (**D**) The box plot shows the correlation between *FMR1* gene expression and pathological GS in PRAD, as well as the Kaplan–Meier survival curves associated with overall survival. * *p* < 0.05, ** *p* < 0.01, **** *p* < 0.0001.

The *FMR1* expression across different pathological stages (pTNM) presented by the violin plots in Figure 3 (left side) shows that the distribution of *FMR1* expression levels varies between the different stages, and the Kaplan–Meier survival curve shows the overall survival and progression-free survival rates for patients in these stages (right side). Our data depict that locally advanced stages (combined pT3 and pT4) exhibit elevated *FMR1* expression levels compared to the localized stage (pT1 and pT2) (*p* = 0.000032). Of interest, patients with localized prostate cancer (pT1 and pT2) have longer overall survival (*p* = 0.012) and progression-free survival (*p* < 0.0001) compared to those with advanced stages (pT3 and pT4) of the disease, as shown by the KM curves in Figure 3A (right side). This is indicated by the survival curves (red and blue lines) remaining highest over the time period, with most patients still alive even after 10 years in terms of overall survival and 15 years in terms of progression-free survival. The pT1 group (red line) has the longest overall survival. The pT2 group (blue line) has intermediate overall survival. Their survival curve is lower than that of the pT1 group but higher than the pT3 + pT4 group. The combined pT3 + pT4 group (orange line) has the shortest overall survival. This group’s survival probability drops significantly earlier and more steeply compared to the other groups (Figure 3A (right side)).

Additionally, patients with regional lymph node metastasis (pN1) shows higher *FMR1* levels compared to those without regional lymph node metastasis (pN0), as determined by the Wilcoxon test (*p* = 0.0038), and as shown by the violin plot in Figure 3B (left side). Similarly, the pN1 group (blue line) shows a drop in overall survival at 5 years that continues to 10 years, while the pN0 group (red line) shows a drop at 10 years that continues to 15 years, as shown in Figure 3B’s KM curves (right side). However, the log-rank *p*-value is 0.107, indicating that the difference is not statistically significant.

The KM curves in Figure 3C show the results of using the best cutoff to separate *FMR1* gene expression (high vs. low) as the main exposure and adjusting for pT2, and the combined pT3 and pT4 tumor stages, which are strong predictors of survival. The high level of *FMR1* expression is associated with short overall survival (*p* = 0.705 and 0.036, respectively). Consistent with this, high levels are also associated with short overall survival in the pN0 (*p* = 0.172), pN1 (*p* = 0.400), and localized M0 (*p* = 0.036) tumor stages.

Our results also indicate that *FMR1* expression is higher in the combined GS 8 and 9 tumors compared to grade 6 and 7 tumors. The box plot in Figure 3D presents the significant differences in expression among these grades. Additionally, when expression levels are split using the best cutoff into high and low groups, high *FMR1* expression is associated with shorter overall survival in the combined advanced-GS group (8 and 9) (*p* = 0.012).

### 2.4. The Influence of the FMR1 Gene on Cancer Pathways Leads to Enhanced PRAD

To understand how the *FMR1* gene might influence PRAD, we examined the relationship between *FMR1* expression data and pathway signatures. The results are summarized in Table 1 and illustrated in Supplementary Figure S1 (plots A through R). We considered the strength and significance of the correlations presented in each plot. The expression levels of *FMR1* show statistically significant positive relationships with critical biological pathways in PRAD. Notably, a moderate positive correlation (*p* = 0.018) between *FMR1* and the cellular response to hypoxia highlights its potential involvement in hypoxic conditions. The G2M checkpoint pathway also shows a strong positive correlation with *FMR1*, suggesting a major role in controlling the cell cycle (*p* <0.00001). In addition, there is a strong link (*p* < 0.00001) between *FMR1* and the *PI3K_AKT_mTOR* signaling pathway, which shows how important *FMR1* is to this pathway. Various findings emphasize the possible connections between *FMR1* and various pathways, indicating opportunities for additional research into their biological consequences. On the other hand, we also found positive but not statistically significant correlations (Table 1). In Supplementary Figure S1, the A, C, G, and Q pathway signatures show weak to moderate positive correlations, but none are statistically significant. D, E, F, and R indicate negative correlations, with none being statistically significant.

Our data indicate a significant positive correlation between the expression of the *FMR1* gene and a substantial regulatory effect on the *PI3K_AKT_mTOR* and G2M checkpoint pathways, which suggests an influential role for *FMR1* in cell division processes.

### 2.5. FMR1 Correlates with PI3K_AKT_mTOR_ Pathway Signature in PRAD

**Figure 4:**
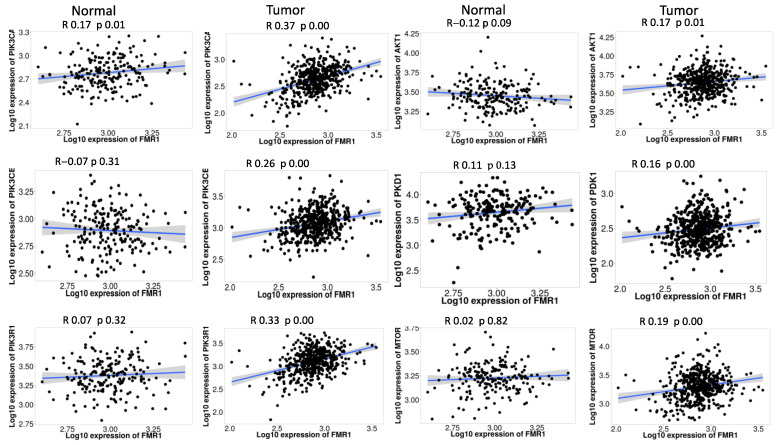
**Correlation analysis of *FMR1* expression with PI3K/AKT/mTOR pathway genes in prostate tissue**. This figure illustrates the correlation between *FMR1* gene expression and genes in the *PI3K/AKT/mTOR* pathway in normal and PRAD samples. The normal panel depicts the correlation in normal prostate samples, while the tumor panel displays the correlation in PRAD samples. Each scatter plot shows the log10 expression of *FMR1* on the *x*-axis against the log10 expression of a specific pathway gene on the *y*-axis, with the Pearson correlation coefficient (R) and *p*-value (p) indicating the strength and significance of each correlation. This analysis shows substantial correlations between *FMR1* expression and various *PI3K/AKT/mTOR* pathway genes, revealing how *FMR1* may affect or be affected by this mechanism in normal and malignant prostate tissues.

We further analyzed the RNA sequencing data to check PI3K_AKT_mTOR pathway genes and *FMR1* in both normal PRAD samples. In normal prostate samples, *FMR1* exhibited a weak positive correlation with *PIK3CA* (R = 0.17, *p* = 0.01) and no significant correlation with *mTOR* (R = 0.02, *p* = 0.82). It is interesting that the link between *FMR1* and *PIK3CA* became stronger in PRAD samples (R = 0.37, *p* < 0.01), which points to a possible connection between *FMR1* expression and *PIK3CA* activation in PRAD. Additionally, we observed a statistically significant but weak positive correlation between *FMR1* and *AKT1* in PRAD samples (R = 0.17, *p* = 0.01), which was not evident in normal samples (R = −0.12, *p* = 0.09). The results also showed a positive correlation between *FMR1* and *PDK1* in tumor samples (R = 0.16, *p* < 0.01) that was not significant in normal samples (R = 0.07, *p* = 0.32) (Figure 4). This indicates a potential modulatory role of *FMR1* in *PDK1* expression during prostate carcinogenesis.

We included *PIK3CB* and *PIK3R1* in the expanded analysis of the *PI3K/AKT/mTOR* pathway’s interaction with *FMR1* gene expression in prostate tissues to provide a more comprehensive view. In healthy/normal prostate tissue, the correlation coefficients for *PIK3CB* (R = −0.07, *p* = 0.31) and *PIK3R1* (R = 0.07, *p* = 0.32) showcase that *FMR1* expression is not significantly related. Both *PIK3CB* and *PIK3R1* have a strong positive correlation with *FMR1* expression in PRAD samples (Figure 4). *PIK3CB* has a correlation coefficient of R = 0.26 (*p* < 0.01), while *PIK3R1* has a stronger correlation with a coefficient of R = 0.33 (*p* < 0.01).

### 2.6. Analysis of FMR1 Copy Number Variations and Their Impact on mRNA Expression in PRAD

**Figure 5:**
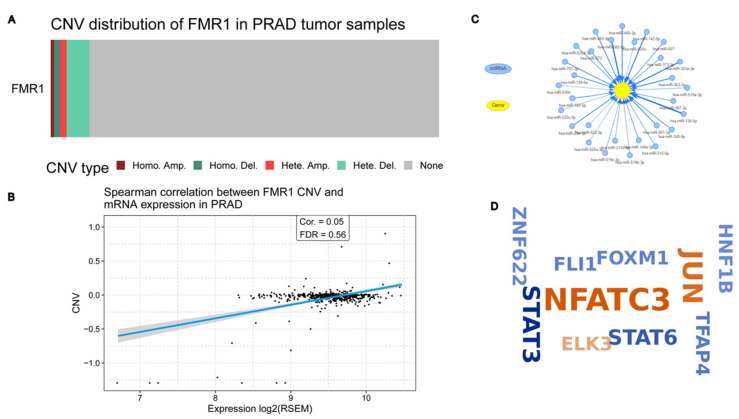
**Unraveling the complex gene regulation mechanisms of *FMR1* expression in PRAD**. (**A**) CNV distribution of *FMR1* in PRAD tumor samples; (**B**) Spearman correlation between *FMR1* CNVs and mRNA expression in PRAD; (**C**) predicted miRNA targets of *FMR1* in PRAD from GSCALite tool; (**D**) predicted TFs that regulate *FMR1* in PRAD were obtained from knockTF tool.

We further explored the *FMR1* CNVs distribution in PRAD tumor samples, which revealed negligible CNVs within the *FMR1* gene. We observed instances of heterozygous deletions and amplifications, but they were relatively infrequent. Notably absent or exceedingly rare were homozygous deletions and amplifications, as presented in Figure 5A.

Additionally, the correlation between CNV and mRNA expression using the Spearman correlation coefficient was 0.05, which means there is not a strong link between *FMR1* CNV and mRNA expression in PRAD. This means there is not any statistical significance. A false discovery rate (FDR) of 0.56 further substantiates this, suggesting that CNV does not significantly modulate *FMR1* expression within PRAD tumors. Figure 5B graphically represents this finding.

### 2.7. MicroRNAs and Transcription Factors Networks Regulating FMR1 Gene Expression in PRAD

Our results revealed a network of miRNAs predicted to target the *FMR1* gene, suggesting an intricate mechanism of post-transcriptional regulation, as shown in Figure 5C. We identified that MiR-323a-3p, among other miRNAs, may also target *FMR1* in PRAD. This discovery aligns with Men’s findings in ESCC, suggesting a potentially broader role for MiR-323a-3p across different cancer types [13]. Finally, we postulate a multitude of transcription factors, such as *JUN, FOXM1*, and *NFATC3*, as regulators of the *FMR1* gene. Figure 5D illustrates the varying influence of these TFs on the gene’s expression using word cloud visualization, where the size of each transcription factor’s representation corresponds to its degree of regulatory impact.

## 3. Discussion

Our investigation provides additional evidence to support the *FMR1* gene’s importance in the field of cancer, beyond its historical significance as a gene initially identified in the context of Fragile X syndrome. In the current study, our data reveal a significant difference in *FMR1* expression between patients with PRAD and the control group (*p*-value = 0.0412), which suggests a potential role of *FMR1* in PRAD. 

Furthermore, higher *FMR1* expression is associated with a higher probability of worse prognosis, evident with disease progression or mortality in patients with PRAD. Surprisingly, the hazard ratio of 5, indicating a high *FMR1* gene expression, may be considered *a* potential risk factor in PRAD. Salem et al. conducted a study that identified six genes associated with colon cancer progression that exhibited high hazard ratios. These genes correlated with an increased risk of colon cancer and worse outcomes [20]. Also, *ME1* gene overexpression was highly related to poor disease-specific and disease-free survival, with hazard ratios of 1.65 and 1.57 [21]. Therefore, a high hazard ratio indicates that *FMR1* may have a role in the pathological process that leads to more aggressive forms of PRAD, as well as serving as a predictor of a worse prognosis.

Patients with the localized PRAD (pT1/2 and pN0) generally have a longer survival time compared to those with locally advanced PRAD (pT3/4 and pN1) [22,23,24]. In our data, the *FMR1* gene is linked to advanced stages of PRAD.

Our results demonstrated substantial stage-specific variation in *FMR1* expression throughout the pTNM PRAD stages. Compared to the localized stages (pT1 and pT2), the expression levels were increased in the later stages (combined pT3 and pT4). The Kruskal–Wallis test verified this with a *p*-value of 0.00032. Overall survival (*p* = 0.012) and progression-free survival (*p* < 0.0001) were significantly longer in localized pT2 patients compared to advanced stages (combined pT3 and pT4). This was reflected in the survival curves, which showed that the majority of patients remained alive over the study period, with survival rates staying high even after 10 years. This is consistent with the expected progression, where earlier stages have better outcomes. S. Lundstam et al. reported that individuals diagnosed with localized renal cell carcinoma (pT1b) demonstrated an increased five-year overall survival rate in comparison to those affected by more advanced stages [25]. Accordingly, *FMR1* expression may be a measure of the severity and progression of the disease.

Furthermore, compared to patients without metastasis (pN0), patients with regional lymph node metastases (pN1) showed significantly elevated *FMR1* levels (*p* = 0.0038). Sood et al., in their study, noted that advanced-stage disease is typically associated with poorer disease-free outcomes [26]. Although the overall survival rate was not statistically significant, the progression-free survival emphasizes the potential of *FMR1* as a biomarker for disease progression in PRAD. Therefore, it may indicate the necessity for larger sample sizes or the inclusion of other factors that influence long-term survival results [27,28]. These results suggest that *FMR1* expression could serve as a predictive tool in clinical practice, but further validation and research are necessary.

In contrast to patients with lower GSs (6 and 7), those with higher grades (combined 8 and 9) exhibited higher levels of *FMR1* expression. In addition, we discovered a correlation between higher levels of *FMR1* expression and decreased overall survival in combined high GSs of 8 and 9 (*p* = 0.012). Guo et al. discovered similar results with different biomarkers, where higher expression levels corresponded with advanced stages and higher GSs, affecting progression-free survival but not overall survival [29]. This study suggests that *FMR1* expression may be a marker for aggressive PRAD prognosis.

GWAS and expression profiling studies demonstrate that the upregulation of this pathway, often due to *PTEN* loss, is a frequent event in PRAD, contributing to tumor formation and resistance to therapy [15]. According to the data we collected, the evidence strongly suggests that the expression level of the *FMR1* gene has a significant positive link with the regulation of the *PI3K_AKT_mTOR* pathway. The cell cycle represented in G2M checkpoint genes also shows a significant positive correlation, indicating that *FMR1* may have a role in regulating cell division processes [30,31,32,33]. The *PI3K_AKT_mTOR* pathway is known for its role in regulating cell growth, proliferation, survival, and angiogenesis, which are all critical processes in the development and progression of cancers, including PRAD [15,16,30]. The significant correlation between *FMR1* gene expression and this pathway suggests that the *FMR1* gene may exert its influence on PRAD development or progression, at least in part, by modulating this pathway. The current report found that *FMR1* gene expression is associated with high-grade GSs and worse overall survival. Additionally, *FMR1* expression is associated with high tumor stages, indicating that higher stages of PRAD are associated with an increase in *FMR1* expression. This finding supports the hypothesis that *FMR1* may play a role in tumor development and may also function as a biomarker for advanced stages of disease. A study of gene expression has also shown that the *PI3K/AKT/mTOR* pathway helps create precursor lesions like high-grade prostatic intraepithelial neoplasia (HGPIN). This demonstrates the significance of this pathway in both the beginning stages of PRAD and its progression [34]. Previous studies have suggested the involvement of various genes in PRAD pathogenesis, but the role of the *FMRI* gene has been less clear [35,36,37,38].

Our data suggest that *FMR1* interacts with *mTOR*, a crucial mTOR complex regulator, significantly impacting cell growth, proliferation, and survival, making it a hub gene in PRAD progression [39]. Moreover, our findings are in agreement with those published by *Shorning* et al., demonstrating that alteration in *PI3K/AKT/mTOR* pathway expression plays a significant role in PRAD development [15]. In line with current tendencies, we found an increase in *PIK3CA* when *FMR1* is present. Both the catalytic subunit p110α and the regulatory subunit *p85α* of *PI3K* are encoded by *PIK3CA*. These subunits have a direct impact on pathway activation and are associated with PRAD development and resistance to therapy [40,41]. In addition, details about the correlation between *FMR1* and *AKT1* encode the *AKT* kinase, which plays a crucial role in various signaling pathways, including those associated with cell proliferation, apoptosis, and glucose metabolism [42]. This pathway activation plays a critical role in cancer [15,43].

These results showed that while *PIK3CB* and *PIK3R1* do not correlate significantly with *FMR1* in normal PRAD tissue, their expression levels become more closely associated with *FMR1* in the context of PRAD. This shift in the correlation pattern may reflect the altered regulatory mechanisms in the tumor microenvironment. The high *p*-values in the tumor samples show how important it might be for these genes to interact with *FMR1* in the disease, pointing to a possible role in the development of tumors or the progression of cancer. In particular, the stronger correlation of *PIK3R1* with *FMR1* could suggest that it is a more influential player in PRAD than *PIK3CB*. Establishing the *PI3K/AKT/mTOR* pathway in expression profiling and the GWAS study greatly improves the findings we acquired by providing a thorough understanding its involvement in PRAD biology.

Given that may influence the *PI3K/AKT/mTOR* pathway, which is involved in cell cycle progression, survival, and metabolism, more in vitro research is required to determine the prognostic significance of *FMR1* in PRAD [44]. These connections make it possible to assume that the *FMR1* gene might impact PRAD by working with and possibly controlling the *PI3K_AKT_mTOR* and G2M checkpoint pathways.

The relationship between miRNA, TFs, and the *FMR1* gene in the context of a disease other than cancer is a complex involving the regulation of gene expression and cellular processes [45,46]. The *FMR1* gene typically shows minimal variation in copy numbers. When variations exist, they do not seem to significantly impact *FMR1*’s mRNA expression levels. This observation suggests that copy number variations may not be critical in regulating *FMR1* expression in PRAD. Instead, regulation might involve a complex interaction with miRNAs and certain transcription factors. Our bioinformatics analyses of miRNAs targeting the *FMR1* gene in PRAD further validate the finding in ESCC, a likely conserved target of MiR-323a-3p in terms of cancer type [13]. While interesting, this form of validation is no substitute for experimental data confirming the interactions and their biological roles. Our finding lays the groundwork for future investigations into the clinical relevance of inhibiting the MiR-323a-3p-*FMR1* regulatory axis in vivo in PRAD. Thus, our study provides insight into the broader context of miRNA, TFs, and their roles in regulation and targeting *FMR1* in PRAD. Prospective studies to further validate the *FMR1* gene as a therapeutic target are needed. Exploring the molecular mechanisms underpinning the function of the *FMR1* gene in PRAD is essential for uncovering new therapeutic opportunities.

## 4. Materials and Methods

### 4.1. Pan Cancer Analysis of FMR1 Gene

RNA-sequencing expression (level 3) profiles for the *FMR1* gene of 33 cancers and normal samples (total 10,228 samples) were analyzed from the TCGA dataset and GTEx, respectively, using the Home for Researchers online public tool (https://www.home-for-researchers.com) (accessed on 1 March 2024). The clinical data included the attributes age, sex, stage of tumor, survival status, and recurrence information. These data are generally tabular CSV (Comma-Separated Values) or TSV (Tab-Separated Values) data.

PanCancer data included ACC (adrenocortical carcinoma), BLC (bladder urothelial carcinoma), BRCA (breast invasive carcinoma), CESC (cervical squamous cell carcinoma and endocervical adenocarcinoma), CHOL (cholangiocarcinoma), COAD (colon adenocarcinoma), DLBC (lymphoid neoplasm diffuse large B-cell lymphoma), ESCA (esophageal carcinoma), GBM (glioblastoma multiforme), HNSC (head and neck squamous cell carcinoma), KICH (kidney chromophobe), KIRC (kidney renal clear cell carcinoma), and KIRP (kidney renal papillary cell carcinoma). These cancers also include LAML (acute myeloid leukemia), LGG (brain lower-grade glioma), LIHC (liver hepatocellular carcinoma), LUAD (lung adenocarcinoma), LUSC (lung squamous cell carcinoma), MESO (mesothelioma), OV (ovarian serous cystadenocarcinoma), PAAD (pancreatic adenocarcinoma), PCPG (pheochromocytoma and paraganglioma), and PRAD (prostate adenocarcinoma). The analysis included READ (rectum adenocarcinoma), SARC (sarcoma), SKCM (skin cutaneous melanoma), STAD (stomach adenocarcinoma), TGCT (testicular germ cell tumors), THCA (thyroid carcinoma), THYM (thymoma), UCEC (uterine corpus endometrial carcinoma), UCS (uterine carcinosarcoma), and UVM (uveal melanoma).

The analyses was conducted using R version 4.0.3. Since the test required was a non-parametric test, a Wilcoxon test was conducted, which does not assume a normal distribution of data. This becomes extremely useful when dealing with non-normal biological data, e.g., gene expression levels.

We used the Wilcoxon test to assess expression differences between tumor tissues and normal tissues, which included 245 nonadjacent tissues and 52 adjacent tissues. The violin plot represents how *FMR1* expression levels are differentially expressed in normal and tumor tissues (in blue and red, respectively), and clearly illustrates the distribution of expression values across these two groups. The normal sample data were obtained from the current-release (V8) GTEx datasets available at the GTEx data portal (https://www.gtexportal.org/home/datasets) (accessed on 1 March 2024).

Overall survival was analyzed across these cancers, and hazard ratios (HRs) were calculated using a univariate Cox regression model provided in the R package. We chose the Cox regression model for its ability to analyze time-to-event data, which revealed a significant association between high *FMR1* expression and shorter overall survival, as indicated by hazard ratios, confidence intervals, and *p*-values displayed in a forest plot. *p*-values less than 0.05 were considered statistically significant (* *p* < 0.05).

### 4.2. Study the Prognostic Impact of FMR1 Gene on PRAD Patients

Once again, we looked into the *FMR1* gene’s predictive value in PRAD samples using the Home for Researchers program. We ranked the samples according to their gene expression levels, using the median as a criterion. In addition to its survival time and status, the figure also shows the relative expression of the *FMR1* gene.

Specifically, in the top scatterplot, blue indicates low expression and red indicates high expression; the scale runs from low to high. The survival status of patients at different time nodes is shown in the middle scatter plot, which is a time-dependent distribution plot. The red dot indicates an alive patient, and the blue dot represents a dead patient. The bottom Figure shows a heatmap of the z-score of *FMR1* expression (blue, low; red, high). A Kaplan–Meier plot shows the time-to-event survival function for patients divided into high-expression and low-expression groups of *FMR1* with a significant difference in overall survival probability (*p* <= 0.05).

HR (high exp) represents the hazard ratio of the low-expression sample relative to the high-expression sample. HR > 1 indicates that the gene is a risk factor, and HR < 1 indicates that the gene is a protective factor. The median survival time (LT50) for both the low and high groups is represented by HR (95% Cl). The receiver operating characteristic (ROC) curve measures how accurately *FMR1* expression levels predict 1-year, 3-year, and 5-year survival outcomes. We implemented the analysis methods and R packages in R version 4.0.3.

### 4.3. Clinicopathology of FMR1 in PRAD

We conducted an assessment of *FMR1* expression in relation to overall survival and disease progression in independent pathological and clinical samples of PRAD patients. The TCGA PRAD dataset has 498 total samples. The TCGA dataset revealed a median follow-up period of 30.5 months for patients undergoing PRAD. Specifically, the median follow-up times are as follows: for overall survival, the median time to event is 36.2 months and the median time to censor is 30.5 months. For progression-free survival, the median time to event is 18.4 months and the median time to censor is 28.2 months [47].

The analysis encompassed the following samples: 476 samples of pathological T, 425 samples of pathological N, and 456 samples of clinical M.

We chose the Kruskal–Wallis test, a non-parametric method, to compare more than two groups because it does not assume a normal data distribution. This enabled the comparison of *FMR1* expression levels across several pathological T stage subgroups, including localized pT1 and pT2, and the combined locally progressed pT3 and pT4, with a significant *p*-value indicating statistically significant differences. We used the Wilcoxon rank-sum test (Mann–Whitney U test) to assess *FMR1* expression between the pathological N-stage subgroups (localized pN0 and advanced pN1).

This test works well with non-normal data distributions, and the significant *p*-value reveals that these groups express *FMR1* differently. Nevertheless, a comparison with the localized M0 subgroup was not practicable because there were only three individuals categorized as metastatic M1 in the clinical M stage. Violin plots display the distribution of *FMR1* expression levels between different pathological stages (localized pT1 and pT2, and locally advanced combined pT3 and pT4) and pathological N stages (localized pN0 and advanced pN1).

We conducted an additional prognosis analysis using the median cutoff, encompassing both the overall survival rate and the prognostic survival rate of different categories. Comparing the survival distributions of two or more groups was accomplished through the use of a log-rank test. The purpose of our application of this test was to determine whether or not there was a correlation between the levels of *FMR1* expression among the disease categories and overall survival. A significant log-rank test result is expected to indicate that high FMRI expression is statistically significant and linked to advanced stages.

Additionally, we used a log-rank test to analyze the prognostic differences in *FMR1* gene expression (high vs. low) as the main exposure, adjusting for tumor stages. Specifically, we tested *FMR1* expression in the pT subgroups (localized pT1 and pT2, and locally advanced combined pT3 and pT4) with high vs. low *FMR1* and evaluated *FMR1* expression in the localized pN0 (high vs. low *FMR1*) and advanced pN1 (high vs. low *FMR1*) subgroups, as well as in the clinical localized M0 subgroup (high vs. low *FMR1*). Although the metastatic M1 subgroup had prognostic information, the small number of cases (only 3) made it challenging to draw reliable comparisons. The Kaplan–Meier survival curves show that a significant log-rank test result supports the statistically significant correlation between high FMRI expression and poor survival in these subgroups’ advanced stages.

We also examined the differences in *FMR1* gene expression among tumors with different GSs and tested the differences in prognostic *FMR1* gene expression (high vs. low) as the main exposure across these GSs. The Kruskal test distribution of *FMR1* expression levels across different GSs (GS 6, GS 7, and GSs 8 and 9 together) is shown in violin plots. We further used a log-rank test to analyze the prognostic differences in *FMR1* gene expression (high vs. low) as the main exposure, adjusting for GS. Specifically, we tested *FMR1* expression for GS 7 and combined GG8 and GG9 with high vs. low *FMR1*. The log-rank test result was used to assess whether there is a statistically significant correlation between high FMRI expression and poor survival at a high-grade GS, excluding GS 6 due to its normality and lack of prognostic information. The Wilcoxon test was applied to test the difference in the expression of the gene in the two groups [48].

### 4.4. Analysis of FMR1 Gene Expression and Pathway Signature Correlations in PRAD

To explore relevant pathways of associated gene sets. We used Home for Researchers webtool feature (accessed on 15 March 2024) to conduct a full analysis using twenty separate scatter plots, labeled A through R, to look at the connections between the levels of *FMR1* gene expression and different biological pathway signatures using the methods from previously published article [49]. Each scatter plot displays data points from different datasets along the X and Y axes. Each graph has the same layout, with the *X*-axis always showing the expression levels that were normalized and log2-transformed (in transcripts per million, or TPM + 1). These levels came from RNA sequencing data. 

These figures represent the expression levels of the *FMR1* gene, as labeled on the axes. The *Y*-axis in each plot refers to a different variable, which changes from one plot to another, covering a variety of biological pathway signatures and processes relevant to our research. 

The single-sample Gene Set Enrichment Analysis (ssGSEA) algorithm’s capacity to assess pathway activity at the single-sample level and the Spearman correlation’s resilience against non-linear and non-normally distributed data were the deciding factors in our choice of these two methods.

We used the GSVA program in R software with ssGSEA as the technique parameter to analyze the data, expecting to find statistically significant associations (*p*-value < 0.05) between *FMR1* expression and pathway activity, suggesting a potential role for this gene in PRAD progression.

### 4.5. FMR1 Gene Expression and PI3K_AKT_mTOR_Pathway Correlations in PRAD

We looked at the link between the expression of the *FMR1* gene and the activity of key genes in the *PI3K_AKT_mTOR*_pathway signaling network using TCGA PRAD data utilizing the TNMplot webtool (accessed on 18 March 2024) [50,51]. The data analysis employed Pearson correlation coefficients to determine the degree of association between the *FMR1* gene expression levels and the expression levels of genes that play a crcial role in the *PI3K_AKT_mTOR* signaling cascade. The statistical significance of these associations was assessed using *p*-value computations, with the significance threshold set at *p* < 0.05. Pearson correlation was chosen for its ability to measure the linear relationship between continuous variables, its suitability for normally distributed log-transformed data, and its straightforward interpretability. The scatter plots show that there is a significant correlation between the log10 expression levels of *FMR1* and PI3K_AKT_mTOR_pathway signaling pathway hub genes. This method allows us to postulate *FMR1*’s potential biological relevance in the context of normal prostate physiology and its dysregulation in PRAD, with a particular focus on its interaction with the *PI3K_AKT_mTOR*_pathway signaling pathway, which is critical for cellular growth and proliferation.

### 4.6. Exploring the Complex Regulation of the FMR1 Gene in PRAD

To evaluate the regulatory factors that impact the expression of the *FMR1* gene in TCGA PRAD, we employed a combination of GSCALite [50,51] and GSCA [20] bioinformatic and statistical analysis tools (accessed on 20 March 2024). We began by analyzing the distribution of copy number variations (CNVs) in *FMR1* using genomic data from PRAD tumor samples. The bar plot illustrates the distribution of CNVs of the *FMR1* gene in PRAD tumor samples. The scatter plot with a linear regression line displays the Spearman correlation between *FMR1* CNV and mRNA expression levels in PRAD. 

To predict potential post-transcriptional regulation, we constructed a network diagram of microRNAs (miRNAs) and transcription factors (TFs) targeting *FMR1*. The interaction network visually represents the interactions between different miRNAs and *FMR1*.

KnockTF 2.0 aims to provide a comprehensive dataset of TF knockdown and knockout experiments across multiple tissue and cell types from different species [52]. This resource facilitates the understanding of TFs’ functions and their regulatory networks by offering extensive data on how the disruption of specific TFs affects gene expression and cellular processes. We used it to predict the TFs that could target and regulate *FMR1* expression in PRAD. The word cloud displays the names of various genes, each represented by a word whose size indicates its significance or relevance in the context of *FMR1*-targeted genes in PRAD.

## 5. Conclusions

The study reveals that *FMR1* gene expression correlates with PRAD progression, with possible links to advanced disease stages and poorer survival rates. This suggests that *FMR1* activity may influence targeted therapies, particularly those involving the *PI3K-AKT-mTOR* pathway. Thus, study also suggests that copy number variations may not be critical in regulating *FMR1* expression in PRAD, but rather, involve a complex interaction with miRNAs and TFs. As a result, our study indicates a possible relationship between miRNA and TFs, their functions in PRAD regulation, and their roles in targeting *FMR1*.

## Figures and Tables

**Table 1:** Correlation between *FMR1* gene expression and molecular signatures in PRAD.

	Pathway	Spearman Correlation Coefficient (r_Spearman)	*p*-Value (p_Spearman)
**A**	Tumor inflammation signature	0.11	0.440
**B**	Cellular response to hypoxia	0.34	0.018
**C**	Tumor proliferation signature	0.22	0.126
**D**	EMT signature	−0.14	0.356
**E**	ECM related genes	−0.29	0.048
**F**	Angiogenesis	−0.01	0.949
**G**	Apoptosis	0.33	0.023
**H**	DNA repair	−0.04	0.806
**I**	G2M checkpoint	0.56	3.55 × 10^−5^
**J**	Inflammatory response	0.10	0.517
**K**	PI3K_AKT_mTOR pathway	0.66	3.53 × 10^−7^
**L**	P35	0.06	0.680
**M**	MYC targets	0.19	0.188
**N**	TGFB	0.44	0.002
**O**	IL_10_Anti-inflammatory signature	0.19	0.187
**P**	Gene upregulated by reactive oxygen (ROS)	0.16	0.263
**Q**	DNA replication	0.23	0.116
**R**	Collagen formation	−0.16	0.290

This table represents different measures of *FMR1* gene expression correlation with various pathways. Positive significance is indicated by a statistically significant *p*-value (*p* < 0.05) and a positive correlation coefficient.

## Data Availability

The data can be obtained from the corresponding author on request.

## Supplementary Materials

The following supporting information can be downloaded at: https://www.mdpi.com/article/10.3390/ijms25137290/s1.

## References

[1] Takahashi S., Takada I. (2023). Recent advances in prostate cancer: WNT signaling, chromatin regulation, and transcriptional coregulators. Asian J. Androl..

[2] Cook D., Sanchez-Carbente M.d.R., Lachance C., Radzioch D., Tremblay S., Khandjian E.W., DesGroseillers L., Murai K.K. (2011). Fragile X Related Protein 1 Clusters with Ribosomes and Messenger RNAs at a Subset of Dendritic Spines in the Mouse Hippocampus. PLoS ONE.

[3] Taylor K.R., Barron T., Hui A., Spitzer A., Yalçin B., Ivec A.E., Geraghty A.C., Hartmann G.G., Arzt M., Gillespie S.M. (2023). Glioma synapses recruit mechanisms of adaptive plasticity. Nature.

[4] Zongaro S., Hukema R., D’Antoni S., Davidovic L., Barbry P., Catania M.V., Willemsen R., Mari B., Bardoni B. (2013). The 3’ UTR of FMR1 mRNA is a target of miR-101, miR-129-5p and miR-221: Implications for the molecular pathology of FXTAS at the synapse. Hum. Mol. Genet..

[5] Hu Y., Gao Q., Ma S., Yu P., Ding S., Yao X., Zhang Z., Lu S., Lu M., Zhang J. (2022). FMR1 promotes the progression of colorectal cancer cell by stabilizing EGFR mRNA in an m6A-dependent manner. Cell Death Dis..

[6] Hanson J.E., Madison D.V. (2007). Presynaptic*Fmr1*Genotype Influences the Degree of Synaptic Connectivity in a Mosaic Mouse Model of Fragile X Syndrome. J. Neurosci..

[7] Zou Z., Wei J., Chen Y., Kang Y., Shi H., Yang F., Shi Z., Chen S., Zhou Y., Sepich-Poore C. (2023). FMRP phosphorylation modulates neuronal translation through YTHDF1. Mol. Cell.

[8] Zhang J., Hou L., Klann E., Nelson D.L. (2009). Altered Hippocampal Synaptic Plasticity in the *Fmr1* Gene Family Knockout Mouse Models. J. Neurophysiol..

[9] Li X.R., Zhou K.Q., Yin Z., Gao Y.L., Yang X. (2020). Knockdown of FBP1 enhances radiosensitivity in prostate cancer cells by activating autophagy. Neoplasma.

[10] Cao H., Gao R., Yu C., Chen L., Feng Y. (2019). The RNA-binding protein FXR1 modulates prostate cancer progression by regulating FBXO4. Funct. Integr. Genom..

[11] Enokida H., Shiina H., Igawa M., Ogishima T., Kawakami T., Bassett W.W., Anast J.W., Li L.-C., Urakami S., Terashima M. (2004). CpG Hypermethylation of *MDR1* Gene Contributes to the Pathogenesis and Progression of Human Prostate Cancer. Cancer Res..

[12] Nianyong Y., Li G. (2023). Comprehensive analysis reveals the involvement of hsa_circ_0037858/miR-5000-3p/FMR1 axis in malignant metastasis of clear cell renal cell carcinoma. Aging.

[13] Men Y., Zhai Y., Wu L., Liu L., Zhang W., Jiang W., Bi N., Song Y., Hui Z., Wang L. (2022). MiR-323a-3p acts as a tumor suppressor by suppressing FMR1 and predicts better esophageal squamous cell carcinoma outcome. Cancer Cell Int..

[14] Morgan T.M., Koreckij T.D., Corey E. (2009). Targeted Therapy for Advanced Prostate Cancer: Inhibition of the PI3K/Akt/mTOR Pathway. Curr. Cancer Drug Targets.

[15] Shorning B.Y., Dass M.S., Smalley M.J., Pearson H.B. (2020). The PI3K-AKT-MTOR Pathway and Prostate Cancer: At the Crossroads of AR, MAPK, and WNT Signaling. Int. J. Mol. Sci..

[16] Bitting R.L., Armstrong A.J. (2013). Targeting the PI3K/Akt/mTOR pathway in castration-resistant prostate cancer. Endocr. Relat. Cancer.

[17] Ding D., Liu G., Gao J., Cao M. (2022). Unveiling the m6A Methylation Regulator Links between Prostate Cancer and Periodontitis by Transcriptomic Analysis. Dis. Markers.

[18] Lucá R., Averna M., Zalfa F., Vecchi M., Bianchi F., La Fata G., Del Nonno F., Nardacci R., Bianchi M., Nuciforo P. (2013). The Fragile X Protein binds m RNA s involved in cancer progression and modulates metastasis formation. EMBO Mol. Med..

[19] Zeng Q., Saghafinia S., Chryplewicz A., Fournier N., Christe L., Xie Y.-Q., Guillot J., Yucel S., Li P., Galván J.A. (2022). Aberrant hyperexpression of the RNA binding protein FMRP in tumors mediates immune evasion. Science.

[20] Baldi S., Zhang Q., Zhang Z., Safi M., Khamgan H., Wu H., Zhang M., Qian Y., Gao Y., Shopit A. (2022). ARID1A downregulation promotes cell proliferation and migration of colon cancer via VIM activation and CDH1 suppression. J. Cell. Mol. Med..

[21] Hu W., Yang Y., Cheng C., Tu Y., Chang H., Tsai K. (2024). Overexpression of malic enzyme is involved in breast cancer growth and is correlated with poor prognosis. J. Cell. Mol. Med..

[22] Kyriakopoulos C.E., Antonarakis E.S. (2017). Surrogate End Points in Early Prostate Cancer Clinical States: Ready for Implementation?. Ann. Transl. Med..

[23] Isbarn H., Huland H., Graefen M. (2013). Results of Radical Prostatectomy in Newly Diagnosed Prostate Cancer. Dtsch. Aerzteblatt Online.

[24] Zapatero A., Banda L.F., Büscher D., Torres L., Conde A.C., Adrados M., Olivier C., Murillo M. (2018). Positive Prostate Biopsy Following Radiation Therapy Can Predict Metastasis-Free Survival In Localized Prostate Cancer. Int. J. Radiat. Oncol..

[25] Lundstam S., Rosenblad A.K., Ljungberg B. (2023). Histopathological upstage, recurrence, and survival in a population-based cohort of surgically treated patients with clinical T1b renal cell carcinoma. J. Clin. Oncol..

[26] Sood A., Rudzinski J.K., Labbate C.V., Hensley P.J., Bree K.K., Guo C.C., Alhalabi O., Campbell M.T., Siefker-Radtke A.O., Navai N. (2024). Long-Term Oncological Outcomes in Patients Diagnosed With Nonmetastatic Plasmacytoid Variant of Bladder Cancer: A 20-Year University of Texas MD Anderson Cancer Center Experience. J. Urol..

[27] Harju S.M., Cambrin S.M., Averill-Murray R.C., Nafus M., Field K.J., Allison L.J. (2019). Using incidental mark-encounter data to improve survival estimation. Ecol. Evol..

[28] Nakajima K., Yamashita T., Kita T., Takeda M., Sasaki N., Kasahara K., Shinohara M., Rikitake Y., Ishida T., Yokoyama M. (2011). Orally Administered Eicosapentaenoic Acid Induces Rapid Regression of Atherosclerosis Via Modulating the Phenotype of Dendritic Cells in LDL Receptor-Deficient Mice. Arter. Thromb. Vasc. Biol..

[29] Guo Y.-S., Wang W.-B., Zhang S.-D., Zhang M.-Q., Qi L., Gao J.-F., Zang Y.-J. (2022). Expression of Glucocorticoid Receptor in Prostate Cancer and Its Clinical Significance. Zhonghua Nan Ke Xue=Natl. J. Androl..

[30] Lozano R., Naghavi M., Foreman K., Lim S., Shibuya K., Aboyans V., Abraham J., Adair T., Aggarwal R., Ahn S.Y. (2012). Global and regional mortality from 235 causes of death for 20 age groups in 1990 and 2010: A systematic analysis for the Global Burden of Disease Study 2010. Lancet.

[31] Carvalho F., Welbourn W., Reid J., Humphreys E., Han M., Lanchbury J., Gutin A., Stone S., Berman D. (2013). 1475 EVIDENCE FOR A CELL CYCLE PROLIFERATION “FIELD EFFECT” IN PROSTATE CANCER. J. Urol..

[32] Stoyanova T., Cooper A.R., Drake J.M., Liu X., Armstrong A.J., Pienta K.J., Zhang H., Kohn D.B., Huang J., Witte O.N. (2013). Prostate cancer originating in basal cells progresses to adenocarcinoma propagated by luminal-like cells. Proc. Natl. Acad. Sci. USA.

[33] van den Hoogen C., van der Horst G., Cheung H., Buijs J.T., Lippitt J.M., Guzmán-Ramírez N., Hamdy F.C., Eaton C.L., Thalmann G.N., Cecchini M.G. (2010). High Aldehyde Dehydrogenase Activity Identifies Tumor-Initiating and Metastasis-Initiating Cells in Human Prostate Cancer. Cancer Res..

[34] Choudhury A.D. (2022). PTEN-PI3K pathway alterations in advanced prostate cancer and clinical implications. Prostate.

[35] Blaine S.M., Honeywell C., Allanson J., Cremin C., Gibbons C.A., Meschino W.S., Permaul J., Carroll J.C. (2009). Genetics Genetics Prostate Cancer. Can. Fam. Physician.

[36] Shand R.L., Gelmann E.P. (2006). Molecular biology of prostate-cancer pathogenesis. Curr. Opin. Urol..

[37] Prasad S., Srivastava S.K. (2019). Mutations in Cancer Driver Genes: An Insight into Prostate Cancer Progression. Ann. Urol. Oncol..

[38] Mazaris E., Tsiotras A. (2013). Molecular Pathways in Prostate Cancer. Nephro-Urology Mon..

[39] Sutherland S.I., Benito R.P., Henshall S.M., Horvath L.G., Kench J.G. (2014). Expression of phosphorylated-mTOR during the development of prostate cancer. Prostate.

[40] Pearson H.B., Li J., Meniel V.S., Fennell C.M., Waring P., Montgomery K.G., Rebello R.J., Macpherson A.A., Koushyar S., Furic L. (2018). Identification of *Pik3ca* Mutation as a Genetic Driver of Prostate Cancer That Cooperates with *Pten* Loss to Accelerate Progression and Castration-Resistant Growth. Cancer Discov..

[41] Alqahtani A., Ayesh H.S.K., Halawani H. (2019). PIK3CA Gene Mutations in Solid Malignancies: Association with Clinicopathological Parameters and Prognosis. Cancers.

[42] Nitulescu G.M., Van De Venter M., Nitulescu G., Ungurianu A., Juzenas P., Peng Q., Olaru O.T., Grădinaru D., Tsatsakis A., Tsoukalas D. (2018). The Akt pathway in oncology therapy and beyond (Review). Int. J. Oncol..

[43] He Y., Sun M.M., Zhang G.G., Yang J., Chen K.S., Xu W.W., Li B. (2021). Targeting PI3K/Akt signal transduction for cancer therapy. Signal Transduct. Target. Ther..

[44] Atkinson S.P. (2021). A preview of selected articles. Stem Cells.

[45] Dolskiy A.A., Yarushkin A.A., Grishchenko I.V., Lemskaya N.A., Pindyurin A.V., Boldyreva L.V., Pustylnyak V.O., Yudkin D.V. (2021). miRNA expression and interaction with the 3′UTR of FMR1 in FRAXopathy pathogenesis. Non-Coding RNA Res..

[46] Anvari M.S., Vasei H., Najmabadi H., Badv R.S., Golipour A., Mohammadi-Yeganeh S., Salehi S., Mohamadi M., Goodarzynejad H., Mowla S.J. (2022). Identification of microRNAs associated with human fragile X syndrome using next-generation sequencing. Sci. Rep..

[47] Liu J., Lichtenberg T.M., Hoadley K.A., Poisson L.M., Lazar A.J., Cherniack A.D., Kovatich A.J., Benz C.C., Levine D.A., Lee A.V. (2018). An Integrated TCGA Pan-Cancer Clinical Data Resource to Drive High-Quality Survival Outcome Analytics. Cell.

[48] Baldi S., He Y., Ivanov I., Sun Y., Feng W., Refat M., Mohammed S.A.D., Adlat S., Tian Z., Wang Y. (2022). Novel characterization discoveries of ferroptosis-associated molecules in COAD microenvironment based TCGA data. Front. Mol. Biosci..

[49] Wei J., Huang K., Chen Z., Hu M., Bai Y., Lin S., Du H. (2020). Characterization of Glycolysis-Associated Molecules in the Tumor Microenvironment Revealed by Pan-Cancer Tissues and Lung Cancer Single Cell Data. Cancers.

[50] Baldi S., Khamgan H., Qian Y., Wu H., Zhang Z., Zhang M., Gao Y., Safi M., Al-Radhi M., Zuo Y.-F. (2021). Downregulated ARID1A by miR-185 Is Associated With Poor Prognosis and Adverse Outcomes in Colon Adenocarcinoma. Front. Oncol..

[51] Baldi S., He Y., Ivanov I., Khamgan H., Safi M., Alradhi M., Shopit A., Al-Danakh A., Al-Nusaif M., Gao Y. (2022). Aberrantly hypermethylated ARID1B is a novel biomarker and potential therapeutic target of colon adenocarcinoma. Front. Genet..

[52] Feng C., Song C., Song S., Zhang G., Yin M., Zhang Y., Qian F., Wang Q., Guo M., Li C. (2023). KnockTF 2.0: A comprehensive gene expression profile database with knockdown/knockout of transcription (co-)factors in multiple species. Nucleic Acids Res..

