# Supplementary figures and images for: The Prognostic and Therapeutic Potential of Fragile X Mental Retardation 1 (FMR1) Gene Expression in Prostate Adenocarcinoma: Insights into Survival Outcomes and Oncogenic Pathway Modulation
Source: Int J Mol Sci. 2024 Jul 2;25(13):7290. doi: 10.3390/ijms25137290 (PMC11242135; doi:10.3390/ijms25137290)

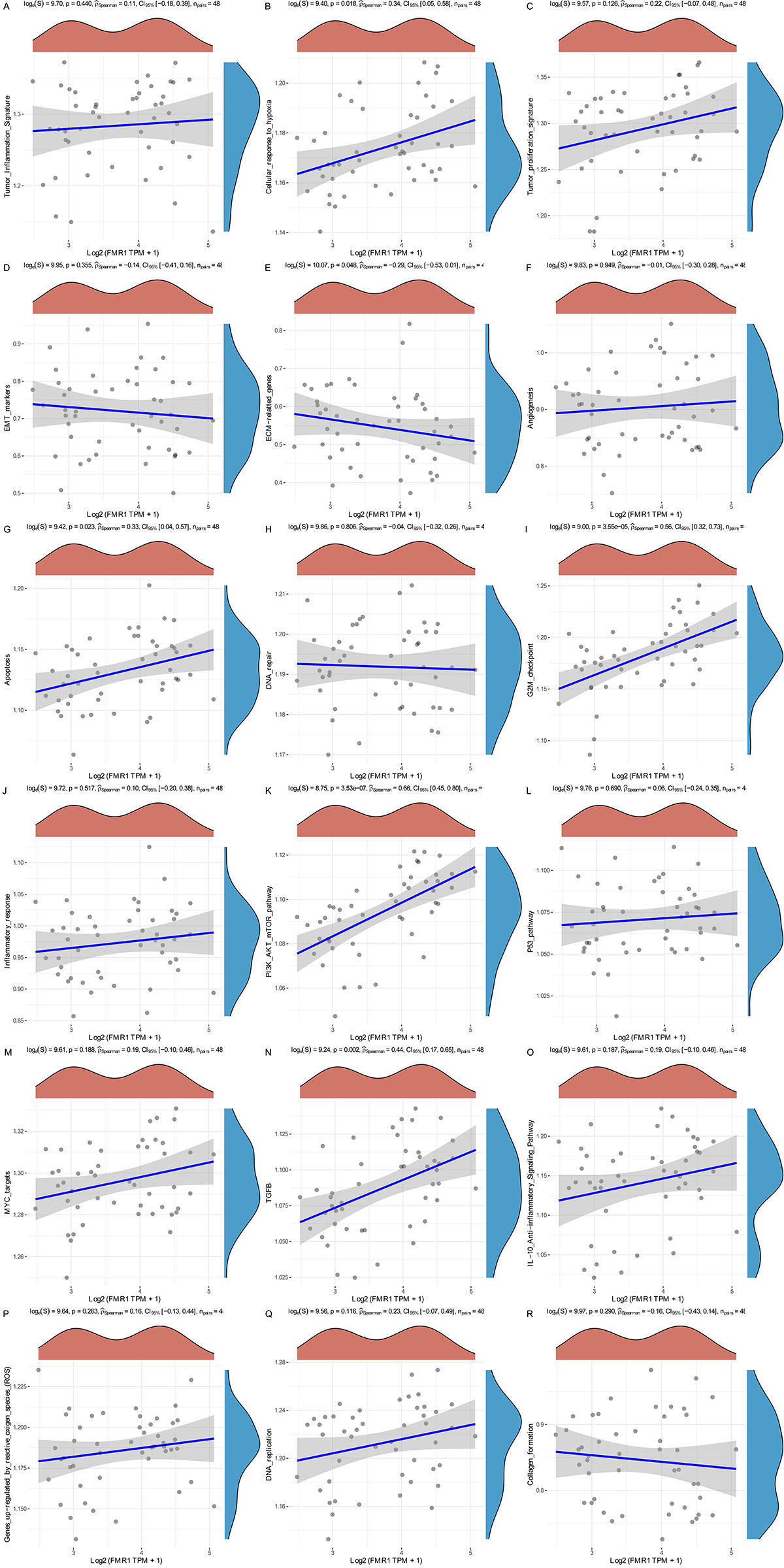

Supplement: Supplementary file 1 [file ijms-25-07290-s001.zip › ijms-3006346-supplementary.tif]
